# Development of nanobodies against the coat protein of maize chlorotic mottle virus

**DOI:** 10.1002/2211-5463.13882

**Published:** 2024-08-21

**Authors:** Faith Njeru, Olivier Zwaenepoel, Geert Haesaert, Gerald Misinzo, Kris De Jonghe, Jan Gettemans

**Affiliations:** ^1^ SACIDS Africa Centre of Excellence for Infectious Diseases, SACIDS Foundation for One Health Sokoine University of Agriculture Morogoro Tanzania; ^2^ Department of Veterinary Microbiology, Parasitology and Biotechnology, College of Veterinary Medicine and Biomedical Sciences Sokoine University of Agriculture Morogoro Tanzania; ^3^ Department of Biomolecular Medicine, Faculty of Medicine and Health Sciences Ghent University Belgium; ^4^ Department of Plants and Crops, Faculty of Bioscience Engineering Ghent University Belgium; ^5^ Plant Sciences Unit Flanders Research Institute for Agriculture, Fisheries and Food (ILVO) Ghent Belgium

**Keywords:** antigen, MCMV, MLN, nanobodies, protein expression, single domain antibody

## Abstract

Maize lethal necrosis (MLN) is a maize disease caused by the maize chlorotic mottle virus (MCMV), a potyvirus which causes yield losses of 30–100%. The present study aimed to isolate nanobodies against the MCMV coat protein (CP) for the diagnosis of MLN. MCMV CP expressed in *Escherichia coli* was used for llama immunization. VHH (i.e. variable heavy domain of heavy chain) gene fragments were prepared from blood drawn from the immunized llama and used to generate a library in *E. coli* TG1 cells. MCMV specific nanobodies were selected by three rounds of phage display and panning against MCMV CP. The selected nanobodies were finally expressed in *E. coli* WK6 cells and purified. Eleven MCMV‐specific nanobodies were identified and shown to detect MCMV in infected maize plants. Thus, our results show that nanobodies isolated from llama immunized with MCMV CP can distinguish infected and healthy maize plants, potentially enabling development of affordable MCMV detection protocols.

## Graphical abstract

Accurate detection of plant diseases is crucial for their effective control, and nanobodies are an attractive alternative for the development of sensitive and accurate diagnostic plant pathogen tools. We developed and tested the use of nanobodies for the detection of maize chlorotic mottle virus, which causes maize lethal necrosis disease.

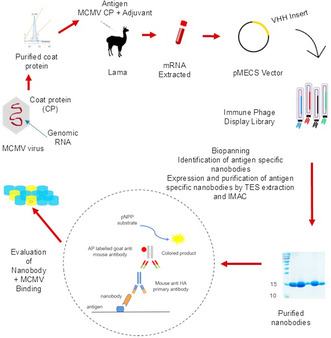

**Abbreviations**
AP: alkaline phosphatase
CP: coat protein
DSMZ: German Collection of Microorganisms and Cell Cultures GmbH
HA: hemagglutinin
IMAC: immobilized metal affinity chromatography
IPTG: isopropyl thio‐β‐d‐galactopyranoside
MCMV: maize chlorotic mottle virus
MLN: maize lethal necrosis
PI: isoelectric pH
SACIDS: Southern African Centre for Infectious Disease Surveillance
SSA: Sub Saharan Africa
VHH: variable heavy domain of heavy chain

In 2012, a devastating maize disease with high infection rates caused by simultaneous infection of maize plants with maize chlorotic mottle virus (MCMV) and any virus in the *Potyviridae* family was reported [1, 2]. This novel disease, identified as maize lethal necrosis (MLN), had previously been documented in Peru in 1973 [3] and in USA in 1979 [4]. Symptoms observed on MLN‐infected plants include mottling, yellowing and dwarfing which vary depending on the age of plant at the time of infection, weather conditions and the maize variety [5, 6]. As a result of the continuous maize cultivation in most regions of Sub Saharan Africa (SSA) and the favorable weather conditions for the vectors, the disease has spread rapidly causing significant yield losses [7]. MLN yield losses range from 30% to 100% [8], posing a significant challenge to maize production in SSA where maize serves as a primary source of food and income.

Sensitive, accurate and rapid detection methods are needed for the prevention and control of plant diseases. In this context, various techniques have been developed to detect MLN causal viruses. These include molecular detection methods that utilize several primers in reverse transcriptase‐PCR and in reverse transcription loop‐mediated isothermal amplification assays to detect MCMV [9, 10]. Although molecular detection is precise, it is not practical for routine diagnosis of large sample volumes because of its high cost and the need for a well‐equipped laboratory.

On the other hand, serological techniques such as an ELISA are suitable for the diagnosis of a large number of samples because of their low cost. However, serological techniques have limitations as they rely on antibodies raised in animals that necessitate multiple validation tests before being approved for certified tests [11]. In addition, the antibodies are huge molecules and cannot be easily engineered for production in large scale in microorganisms [12]. Antibodies also require complex eukaryotic expression systems and they cannot be easily conjugated to other molecules [13].

However, Camelidae serum have distinctive antibodies known as heavy chain antibodies that differ from conventional immunoglobulins by lacking the light chain [14]. The antigen binding region of these antigens consists of a single domain, which allows for easy production in a recombinant form in microorganisms. This single domain antibody is referred to as VHH (i.e. variable heavy domain of heavy chain) or nanobody [15]. Nanobodies can withstand high temperatures and alkaline conditions, making them suitable capture agents for immunodetections [16].

Nanobodies have been successfully isolated and applied for detection of plant pathogens. For example, a study [11] isolated nanobodies for the detection of *Tulip virus X*. Additionally, nanobodies have been developed to detect tomato leaf curl Sudan virus, the most prevalent begomovirus in Saudi Arabia [17]. Furthermore, nanobodies can be conjugated to fluorescence and other molecules for other applications [13]. For example, nanobodies linked to enhanced green fluorescence protein and alkaline phosphatase were used to develop a sandwich ELISA for detecting Mal de Rio Cuarto virus [18].

Target specific nanobodies are retrieved from either immune, synthetic or naïve nanobody libraries [19]. Immune libraries are preferred because they produce affinity matured nanobodies. The attainment of immune nanobody libraries requires the immunization of animals with properly folded recombinant protein [20]. Therefore, the present study aimed to leverage the unique properties of nanobodies and evaluate their effectiveness in detecting MCMV, comprising the major causative virus for MLN.

## Materials and methods

### MCMV coat protein (CP) gene

The CP gene sequence for MCMV were retrieved from the GeneBank under accession number MK491606. Fifteen nucleotides were added at 5′ and 3′ for cloning based on homologous recombination. The designed sequences, gBlock MCMV CP gene (Fig. S1), were ordered from Integrated DNA Technologies, Inc. (Coralville, IA, USA) and used for downstream processes.

### Cloning MCMV gBlock sequences to a prokaryotic expression vector pTYB12

Using the cold fusion method, the gBlock sequences were cloned to the pTYB12 vector. The pTYB12 vector was linearized by restriction enzyme digestion using the *Eco*R1 and *Nde*l enzymes. The digestion reaction was done at 37 *°*C for 3 h and the digest was purified using the PCR clean‐up kit (Machery‐Nagel, Duren, Germany) in accordance with the manufacturer's instructions. Cold fusion cloning was performed where 50 ng of the linearized vector was mixed with 100 ng of the synthesized sequences (inserts), incubated at room temperature for 5 min and then placed on ice for 10 min to allow the insert to fuse to the linearized vector. The cloning mixture (recombinant plasmid) was then used for transformation experiments.

### Transformation

In total, 50 μL of cold fusion competent cells were added to 10 μL of the cloning mixture and transformation by heat shock was carried out following the laboratory set procedure. Briefly, the competent cells were mixed with cloning mixture and allowed to incubate on ice for 20 min, then the heat shock was performed for 50 s at 42 °C and the bacteria cells immediately put back on ice for 2 min. Afterwards, super optimal broth with catabolite repression medium was added and the cells were grown for 1 h at 37 °C to allow expression of the ampicillin resistance gene. In total, 150 μL of the transformation mix was plated on agar plates containing 50 μg·mL^−1^ ampicillin and allowed to grow overnight at 37 *°*C. Colonies were tested for the presence of the insert with the *Eco*R1/*Nde*l test digest after plasmid preparation and run on 1% agarose gel in Tris‐acetate EDTA buffer. Colonies with the correct insert were confirmed by DNA sequencing. After sequence alignment, clones with the correct sequence were identified and used for expression studies.

### Expression of MCMV CP fused to the intein tag

The plasmid with the correct target sequence was used for the expression studies. The plasmid was transformed into *Escherichia coli* BL21 DE3 competent cells by the heat shock method (see above). The transformed cells were plated onto agar plates containing 50 μg·mL^−1^ ampicillin and incubated overnight at 37 *°*C. A single colony was grown overnight in 10 mL of LB medium (10 g tryptone, 5 g of yeast extract, 10 g NaCl and 1 L of distilled water with pH adjusted to 7.0 before autoclaving the mixture for 25 min at 120 °C) supplemented with 50 μg·mL^−1^ ampicillin to produce a starter culture used to inoculate 1 L of Terrific broth medium. The expression of the recombinant protein was induced once *D*_600_ of 2.0 was reached by the addition of 1 mm isopropyl thio‐β‐d‐galactopyranoside (IPTG). The cultures were incubated overnight at 20 °C in a shaking incubator.

### Cell lysis and purification of MCMV coat protein

After the overnight incubation, the cultures were centrifuged at 6000 ***g*** for 15 min at 4 °C in a Sorval instrument (Thermo Fisher Scientific, Waltham, MA, USA) and the pellet was resuspended in chitin column buffer (20 mm Tris‐HCl, pH 8.5, 500 mm NaCl, 1 mm EDTA). The cells were lysed by passing them twice through a French press, followed by a brief sonication to shear genomic DNA. Subsequently the lysate was centrifuged at 10 000 ***g*** for 30 min at 4 °C (Sorval, Zevenhuizen, The Netherlands) and the supernatant was loaded onto a column containing 5‐mL chitin beads (New England Biolabs, Ipswich, MA, USA) to bind the intein fusion protein. The lysate was run through the column at a flow rate of 1 mL·min^−1^ at 4 °C. The column was washed with the chitin column buffer to remove aspecific binders, then flushed with 50 mm dithiothreitol and the flow stopped before the beads run dry and left overnight to allow cleavage. The protein was eluted in 0.5‐mL fractions with 50 mm dithiothreitol dissolved in 20 mm Tris‐HCl, pH 8.5, 500 mm NaCl and 1 mm EDTA at room temperature. Fractions were analysed by SDS/PAGE. The eluted proteins were dialysed to a buffer with lower salt concentration (buffer A composed of 20 mm Tris, pH 8.0, 1 mm EGTA, 50 mm NaCl and 0.5 mm dithiothreitol) and further purified by ion exchange chromatography. The dialysed proteins were centrifuged for 10 min at 14 000 ***g*** at 4 °C to remove any possible precipitation and loaded on MonoS 5/5 column (9644021; Pharmacia Uppsala, Sweden). The samples were allowed to bind and the column was washed with buffer A to remove contaminants. The protein of interest was then eluted in a salt gradient (20 mm Tris, pH 8.0, 1 mm EGTA, 1 m NaCl and 0.5 mm dithiothreitol). The eluted protein was dialysed again in buffer A and the purity assessed by SDS/PAGE and Coomassie blue staining.

### Llama immunization

A healthy male llama was immunized four times over a period of 6 weeks with each immunization involving the administration of 250 μg of the purified CP. The immunizations were performed on days 0, 14, 28 and 35. Peripheral blood was drawn from the llama before immunization and after the last immunization was performed to monitor the progress of the immunization.

The immunization was conducted at CER groupe (Marche‐en‐Famenne, Belgium), a Walloon government funded animal facility where the llama was kept, by an authorized veterinarian. The proper ethical guidelines for conducting animal research as set by the EU 2010/63 directive for the protection of animals for scientific purposes were followed. In addition, the guidelines set by the ethics committee and the animal welfare body for Ghent University in the Faculty of Medicine and Health Sciences were followed. The protocol used for llama immunization was reviewed and approved by CER Groupe's ethical committee within the project ‘CE/Santé/E/001 V2: hyperimmunization en vue de la production d'anticorps polyclonaux’.

### Immune library construction

After the final injection, 100 mL of blood was drawn from the llama. The blood was diluted in equal volume of 0.9% sodium chloride and transferred to Leucosep tubes (Greiner Bio‐One, Vilvoorde, Belgium). Peripheral blood lymphocytes were purified from the diluted blood by centrifugation. MCMV Nb library was constructed following the protocol described by Vincke *et al*. [21]. In brief, total RNA isolated from the peripheral blood lymphocytes was used as template to synthesize cDNA. The cDNA was subsequently used to amplify for the VHH genes in a nested PCR protocol using target specific primers. CALL001 and CALL002 primers were used for the first PCR, whereas VHHBACK and PMCF primers were used for the second PCR [21]. The purified PCR products were digested with *Pst*1 and *Not*1 restriction enzymes and ligated in frame and downstream of a pelB leader sequence, and upstream of a hemagglutinin (HA) tag, His_6_ tag and the M13 gene 3 in pMECS vector. *E. coli* TG1 cells were transformed with the purified ligation mixture and plated on selective media to generate MCMV VHH library [21].

### Retrieval of MCMV CP specific nanobodies

A representative aliquot of the library was grown in 2XTY/Glu‐AMP media, the cells were then infected with M13K07 helper phages to produce phage particles displaying the nanobody protein at their tip. The resulting virions were used for panning against the immobilized MCMV CP recombinant antigen that was coated on 96‐well plate [15]. The virions eluted from the immobilized antigen by addition of triethlyamine were subsequently used to infect fresh *E. coli* TG1 cells, whereas others were used to evaluate for enrichment of antigen specific nanobodies on LB‐AMP/GLU agar plates. The panning process was repeated three times. The presence of MCMV specific nanobodies was tested in an ELISA using the periplasmic extracts from the *E. coli* TG1 cells infected with dilutions of the antigen eluted phage particles and plated on selective agar plates [21]. After the periplasmic ELISA, 32 positive colonies were randomly selected and used in colony PCR to calculate the percentage of colonies with an insert the size of VHH. In addition, selected samples that were positive for the correct VHH length after the colony PCR were sent for Sanger sequencing. A phylogenetic tree was drawn in mega11 (https://www.megasoftware.net) from the translated amino acid sequences; the sequences were aligned in clustal w (https://www.genome.jp/tools‐bin/clustalw) and saved in Fasta format. The phylogenetic tree was constructed using maximum likelihood statistical method with the poisson model. Bootstrap analysis with 1000 replicates was performed to evaluate the significance of the interior branches. For the other parameters the default settings were used.

### Expression of MCMV specific nanobodies

Eleven clones were selected and the pMECS‐Nbs plasmid isolated using a plasmid mini prep (Macherey‐Nagel) in accordance with the manufacturer's instructions. For nanobody expression, WK6 *E. coli* cells were used because it comprises a non‐suppressor strain and an amber stop codon exists between the Nb and the phagemid gene 3, allowing the expression of only the nanobody gene. Therefore, WK6 *E. coli* cells were heat‐shock transformed with pMECS‐Nb plasmids. The transformed cells were allowed to grow in TB at 37 °C until a *D*_600_ of 0.8 was reached, after which IPTG was added to induce protein expression and the cultures were incubated overnight at 28 °C. To retrieve the nanobodies expressed in the periplasm of the cells, the cells were pelleted after overnight incubation and subjected to an osmotic shock using Tris‐EDTA‐Sucrose (TES, 0.2 m Tris, 0.5 mm EDTA and 0.5 m sucrose, pH 8.00) [21]. The TES extracts were further purified by immobilized metal affinity chromatography (IMAC) because the expressed nanobodies are fused to HA tag and His_6_ tag at their C‐termini. Protein concentration of nanobodies was measured using the Bradford assay (Bio‐Rad Laboratories, Hercules, CA, USA) and SDS/PAGE was used to analyse their quality.

### Binding ability of the MCMV nanobodies

The binding ability of the nanobodies was evaluated against MCMV infected maize plants through an ELISA assay. MCMV infected maize plants to be used as biological samples were obtained through mechanical inoculation of 3–4‐week‐old maize plants, which were sown in a greenhouse compartment with GL‐Q (quarantine) containment level at the Flanders Research Institute for Agriculture, Fisheries and Food (ILVO, Merelbeke, Belgium). This aimed both to prevent MCMV ‘escaping’ the research facilities, as well as to prevent other viruses infecting the maize plants. The inoculum source was taken from other maize plants, mechanically infected with MCMV isolate PV‐1087 (GenBank accession OK181780) obtained from the German Collection of Microorganisms and Cell Cultures GmbH (DSMZ) (Braunschweig, Germany), yet originating from a natural maize infection in Kenya. The infection was checked both by ELISA (Antibody set RT‐1087, directly developed against isolate PV‐1087, and also obtained from DSMZ), as well as by real‐time PCR, using the primers/probe and procedure reported by Adams *et al*. [22]. These infected plants (Fig. S2) served as MCMV source for the ELISA.

### Indirect enzyme linked immunosorbent assay (indirect‐ELISA)

The purified nanobodies were tested for their ability to recognize MCMV using the indirect ELISA where MCMV inoculated leaf samples were used. Only six of the 11 selected nanobodies were used in the indirect ELISA. The nanobodies were selected from each family group and also depending on the expression levels as observed on the SDS/PAGE gel. Briefly, a 96‐well plate was coated overnight with 100 μL of the infected leaf samples ground in phosphate buffer. After the incubation, the wells were blocked by addition of a blocking solution (phosphate buffered saline with 1% milk), washed and then incubated with the nanobody for 2 h. Mouse anti‐HA antibody, comprising a monoclonal antibody obtained from ROTH (Karlsruhe, Germany), was added followed by goat anti‐mouse antibody conjugated with alkaline phosphatase (AP), a polyclonal antibody (ROTH). *p*‐Nitrophenyl phosphate, disodium substrate solution was added and color development was allowed to occur, after which absorbance was measured at 415 nm in a plate reader. A bar chart was drawn in r studio (https://posit.co/products/open‐source/rstudio) to visualize and analyse the indirect ELISA results [23].

### Antibodies

Mouse anti‐HA antibody and goat anti‐mouse AP used in the indirect ELISA assay were obtained from ROTH and they are produced for research purposes only. Mouse anti‐HA antibody is a monoclonal antibody from the clone 12CA5, whereas goat anti‐mouse AP is a polyclonal antibody that is affinity purified.

The RT‐1087 antibody set is a polyclonal antibody supplied as purified IgG for research purposes only and was obtained from DSMZ.

## Results

### Transformation of the plasmid to bacterial cells

**Fig. 1:**
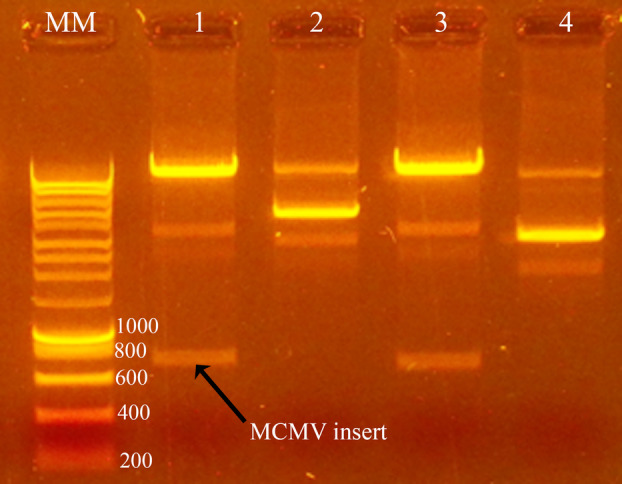
*Eco*R1/*Nde*l test digest after plasmid preparation for MCMV CP constructs run on 1% agarose gel. MM is the molecular marker in base pairs (bps), while lanes 1, 2, 3 and 4 are the samples. The correct insert size of approximately 750 bp was observed on constructs loaded on lanes 1 and 3.

gBlock MCMV CP was successfully cloned to the pTYB12 vector. Colonies with the insert of interest (MCMV, 754 bp) were confirmed on 1% agarose gel after the *Eco*R1/*Nde*l restriction enzyme digest. Samples 1 and 3 had the bands of the expected length for gBlock MCMV coat gene (Fig. 1). Analysis of the Sanger sequencing results (Fig. S1) confirmed that the positive constructs had identical match to the original vector (sequence plus the insert coding sequence in frame with the intein sequence).

### Expression and purification analysis of MCMV coat protein

**Fig. 2:**
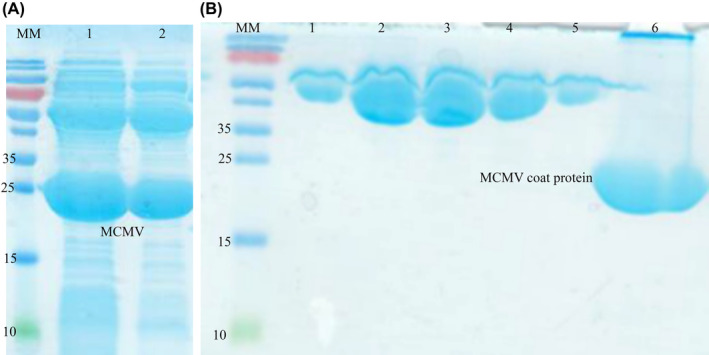
Expression of MCMV CP in *E. coli* BL21 DE3 cells. Protein purification was achieved by chitin column affinity purification and MonoS ion exchange chromatography. (A) 15% SDS/PAGE was used for analysis after chitin column affinity purification of MCMV coat protein. The gel lanes were loaded as follows: MM, molecular weight marker in kDa; l and 2; products after the overnight self‐cleavage induced by dithiothreitol. MCMV CP was observed at 25 kDa. From the gel image, other proteins co‐eluted with the desired protein. (B) 15% SDS/PAGE analysis after MonoS ion purification of MCMV coat protein, with the protein of interest eluted using NaCl salt gradient. MM is the molecular weight marker; 1–6 are the fractions eluted during the chromatographic process. 1 to 5 are the eluted NaCl gradient fractions. MCMV CP eluted when the concentration of NaCl was at 1 m.

MCMV CP was expressed from 1 L of bacterial culture in *E. coli* BL21 DE3 after induction with 1 mm IPTG as a fusion protein, joined to the intein tag. In SDS/PAGE analysis, the cleaved protein was obtained in a soluble form with a predicted molecular mass of 25 kDa following the chitin column affinity purification protocol (Fig. 2A). In addition, it was observed from the gel image that the eluted fraction also contained some other unwanted proteins (Fig. 2A). Because purified protein antigens are preferred for immunization [24], the obtained MCMV CP fractions were subjected to further purifications by ion exchange chromatography. MCMV CP isoelectric point was approximately 10; therefore, a MonoS cation exchanger was used for protein purification. MCMV coat protein eluted at 0.3 m NaCl (Fig. S3). According to the SDS/PAGE analysis, the MCMV coat protein was judged to be highly pure with no major impurities from *E. coli* (Fig. 2B), allowing us to generate a good‐quality antigen for llama immunization. The protein obtained had a concentration of 1.3 mg·mL^−1^.

### Assessment of llama immune response to MCMV CP

**Fig. 3:**
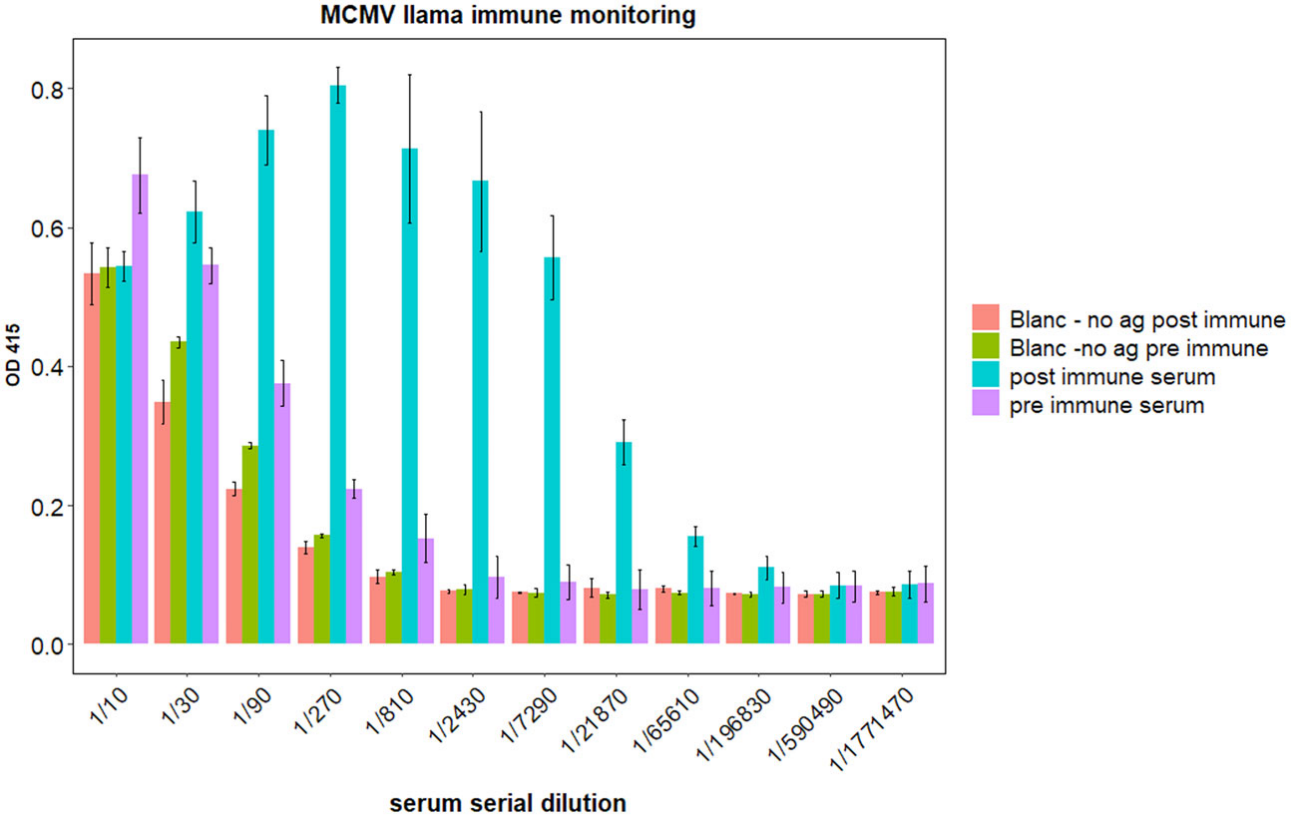
Representative immune monitoring showing the strong immune response to MCMV coat antigen. (in this response measure, both conventional and heavy chain antibodies are measured). The serum of the llama from the blood drawn before and after immunization was serially diluted from 1 : 10 up to 1 : 1 771 470 (*n* = 12 for each dilution loaded in triplicate, geom_bar drawn using ggplot2 with the SD used to indicate the error bars).

The MCMV capsid is a protein that is composed of 180 copies of biochemically identical coat protein. In this study, the coat protein was produced in a recombinant form as explained above and used as the antigen for immunization. To generate nanobodies specific for MCMV CP, a healthy llama was immunized with the CP antigen following proper animal handling procedures. The immunized llama developed a robust immune response against MCMV CP as shown by the ELISA results after analysis of the sera obtained from test bleeds at pre‐immune and post‐immune time points (Fig. 3). The antibodies in the llama serum, diluted up to 1.8 × 10^−6^, could still detect the MCMV CP.

### Selection for MCMV specific nanobodies

**Fig. 4:**
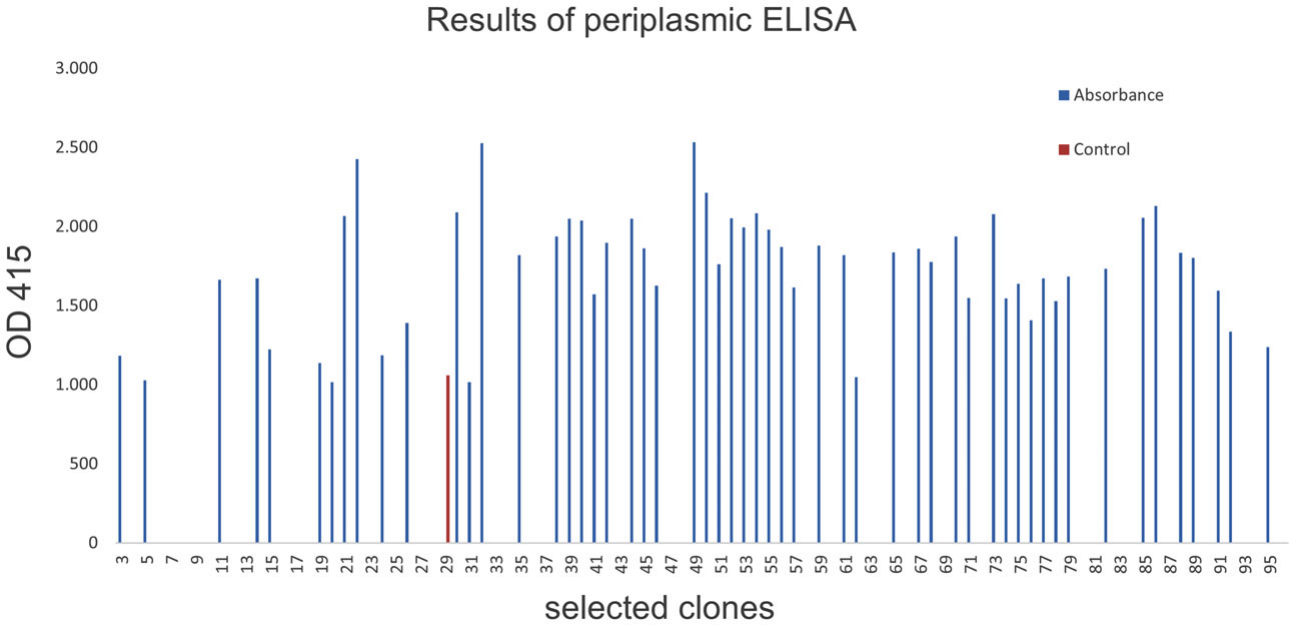
Identification of MCMV CP specific nanobodies by periplasmic ELISA. From the analysis of 95 clones randomly picked from the second and third round of panning, 76 clones were identified as positive for nanobodies that bind MCMV CP. The absorbance read at 415 nm was at least twice in the antigen coated well compared to the control well. However, clone 29 had a higher reading in the control well, which could be a result of contamination.

**Fig. 5:**
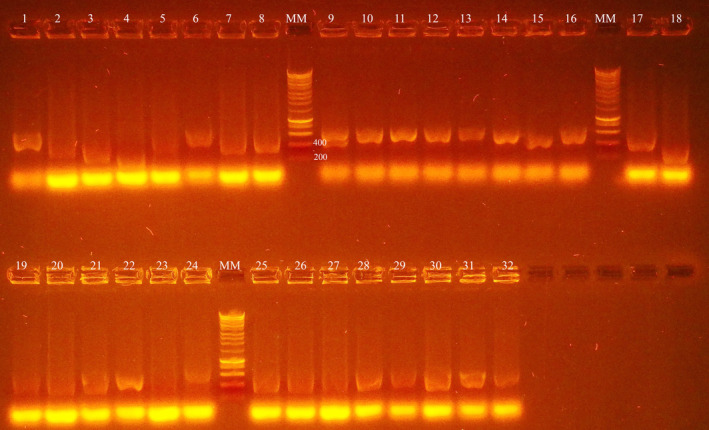
Colony PCR to test for colonies containing an insert with the desired MCMV VHH genes. The PCR products were analyzed on a 1% agarose gel. A positive nanobody clone produces an amplicon of approximately 400 bp. MM is the molecular marker in base pairs (bps) while 1 to 32 are the samples. Of the 32 selected clones, 28 of them had a fragment that was approximately 400 bp.

**Fig. 6:**
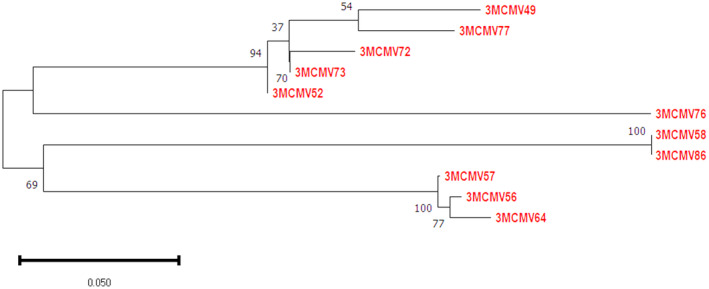
Phylogeny tree of the protein sequences of the selected nanobodies demonstrating the homology between the different VHH sequences against the MCMV CP. The sequences group to four families.

Total RNA isolated from the blood of the immunized llama was used to construct an immune VHH library in pMECS containing 7.7 × 10^7^ transformants. MCMV CP specific nanobodies were selected from the generated immune library by three consecutive rounds of solid phase *in vitro* selection in ELISA plates coated with recombinant MCMV CP. Ninety‐five clones were randomly picked from colonies from the second and third round of panning to select for nanobodies against MCMV CP by periplasmic ELISA. The results of the periplasmic ELISA showed that 76 of the selected colonies were positive (expressing a nanobody that was recognizing the MCMV CP antigen) (Fig. 4). The positive colonies were selected that had an absorbance in the antigen coated well that was at least twice that of the well without the antigen for the same periplasmic extract (Table S1). Of the clones with a positive signal, 32 were randomly selected for colony PCR. Colony PCR analysis demonstrated a frequency of clones having a VHH insert of 87.5% (Fig. 5). Eleven of the selected nanobodies were sequenced using the Sanger sequencing method and the sequences translated to amino acid sequences using the translate tool in expasy (https://web.expasy.org/translate). The selected sequences grouped to four families as shown from the phylogenetic tree (Fig. 6).

### Nanobody expression and purification

**Fig. 7:**
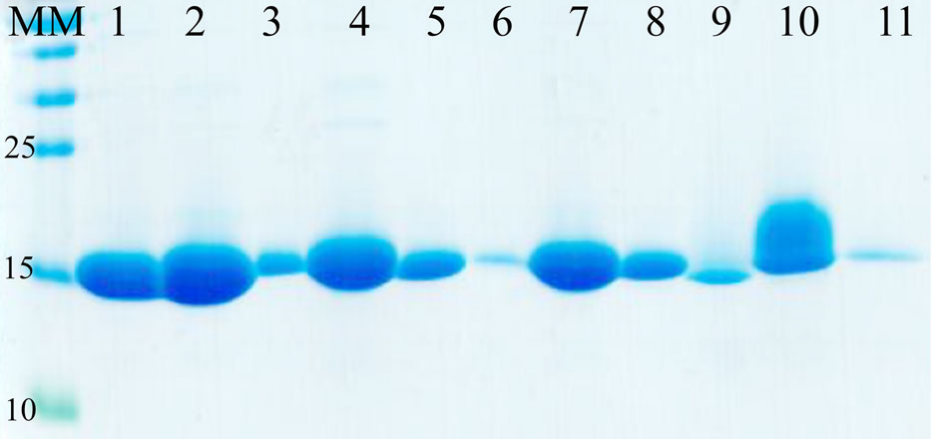
Soluble expression of MCMV CP specific nanobodies in *E. coli* WK6 cells. Protein purification was achieved after osmotic shock lysis and IMAC purification. In the first well, MM is the molecular marker and the nanobodies were loaded in the remaining wells. A good quality band of approximately 15 kDa is observed on 15% SDS‐PAGE gel indicating high quality nanobodies.

To express the nanobodies, the 11 recombinant pMECS vector containing the Nb genes were introduced to competent WK6 *E. coli* cells by heat shock at 42 °C. The expressed nanobodies were soluble and had a molecular weight of about 15 kDa as shown on SDS/PAGE. In SDS/PAGE, all the 11 selected nanobodies were of good quality and high purity with less contaminants (Fig. 7). After measuring the concentration of the nanobodies using the Bradford protein assay, an average concentration of 3.3 mg·L^−1^ was recorded.

### Characterization of the nanobodies

**Fig. 8:**
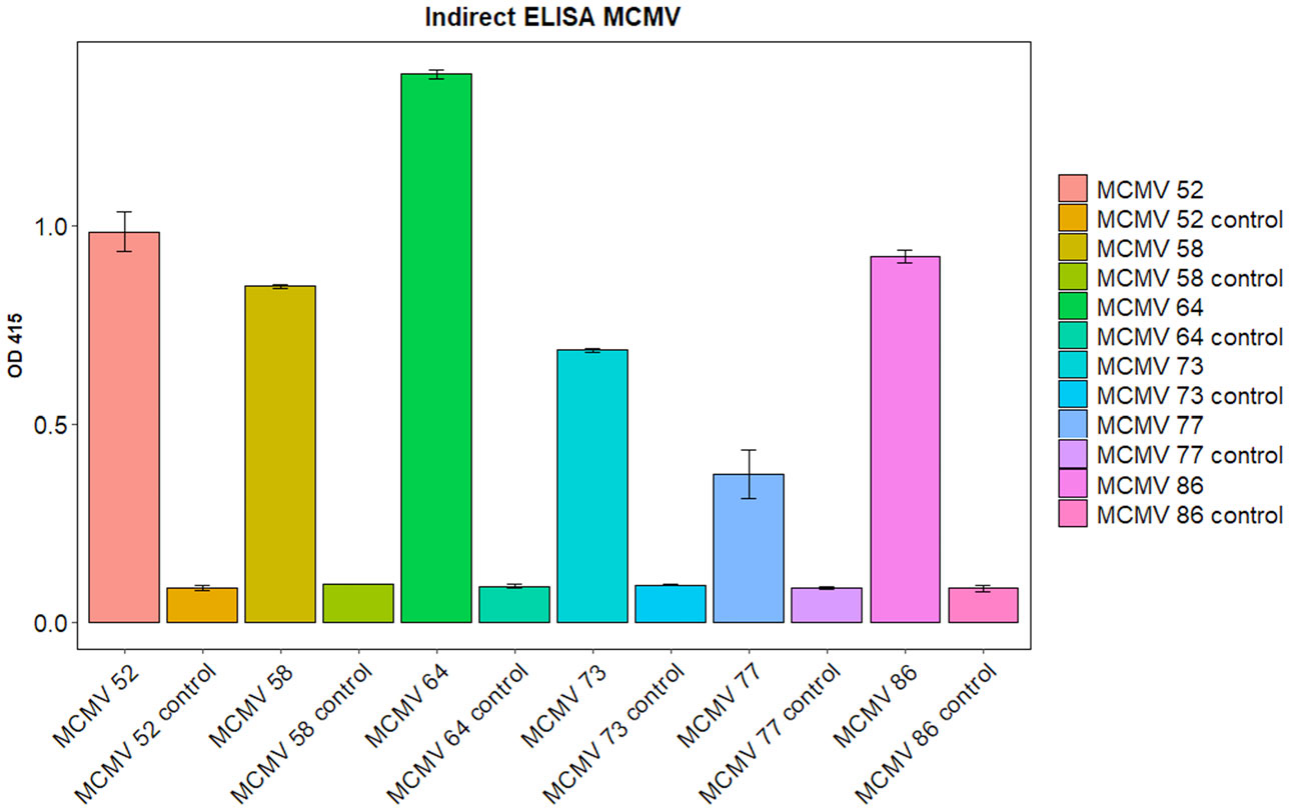
A bar chart representing absorbance at 415 nm of the nanobodies developed against MCMV CP in indirect ELISA. The test wells were coated with 100 μL of sample material prepared from MCMV infected maize plants. The control wells were coated with extraction buffer. All six tested nanobodies were detecting MCMV antigens with a high signal compared to the control well (*n* = 17 loaded in duplicate, geom_bar drawn using ggplot2 with the SD used to indicate the error bars).

The feasibility of the nanobodies to bind to MCMV in infected maize samples was tested by the indirect ELISA. All six selected nanobodies against MCMV CP demonstrated clear rapid detection signals on ELISA plate upon development because the absorbance reading of the ELISA results was greater than 2 × negative control (Fig. 8). The nanobody labeled as MCMV 64 had the best binding signal compared to the other nanobodies.

## Discussion

Nanobodies, which are also known as VHHs, are antibody fragments derived from heavy chain only antibodies found in the serum of camelids [13]. Subsequent to the first report on nanobodies in 1993, they have steadily gained attraction as a research tool in diagnostics because they can be produced easily in microorganisms using recombinant technologies [25]. Nbs are monomeric and are the smallest (15 kDa) antibody fragments that maintain the specificity and sensitivity of antigen binding of the original whole antibody [26]. They are highly stable and can be easily engineered to complex moieties for various applications [27]. Immune libraries are preferred for generation of target‐specific Nbs because they generate antibody libraries that are highly specific and have a greater affinity to the antigen of interest [28].

Several studies have explored the generation of nanobodies against different antigenic targets. One example is nanobody 122, developed from an immunized alpaca against recombinant interferon protein demonstrating efficacy in ELISA analysis. When coupled with colloidal gold nanoparticles, this nanobody was used to develop a lateral‐flow immunochromatographic test strip with a limit of detection of 1 μg·mL^−1^. Compared to traditional antibodies, nanobody 122 proved to be more stable, sensitive and inexpensive, offering an alternative way for developing diagnostic tests [29]. In another study, a highly sensitive sandwich ELISA using nanobodies as capture and detecting agents has been optimized for detection of Mal de Río Cuarto virus, a *Reoviridae* virus that severely affects maize [18]. Additionally, a nanobody based antigen detection lateral flow assay with a 92% specificity was developed for identifying *Trypanosome congolense* [30].

Because MLN is a relatively new disease on the African continent, very few strategies are available for its control [2]. Integrated pest management, which includes early disease detection, is an important tool that can contribute to the management of MLN. Besides early detection being key in integrated pest management, MLN has been declared a quarantine pathogen in Kenya and several other countries in SSA, necessitating the testing of maize samples for export and import. Given the promising results of previous studies on nanobody based detection protocols, the present study aimed to develop nanobodies against MCMV, one of the main causal viruses for MLN, and to evaluate their effectiveness for disease detection.

MCMV (754 bp) coat protein cDNA was cloned to the pTYB12 vector and expressed. The recombinant protein was further purified using ion exchange chromatography. The isoelectric pH (PI) for MCMV coat protein was 10.45, which was estimated after computation in expasy (https://web.expasy.org/protparam) This estimated PI was greater than the pH of the buffer (pH 8); therefore, monoS (cation exchange) chromatography was used for purification. Fortuitously, a single protein band (25 kDa) was observed on SDS/PAGE and Coomassie blue staining following MonoS purification.

Although most studies on plant viruses [31] use the purified viral particles as the antigen, the present study used the recombinant coat protein. Interestingly, from the immunized llama, we were able to generate a good immune library of 10^7^ transformants in which more than 80% of the clones had a plasmid with a VHH insert. These results therefore show that it is possible to generate nanobodies against plant viruses from the recombinant coat protein without having to use the purified virus particles that require more expertise to purify.

The phage panning screening process, which was performed three consecutive times, yielded several clones and the 11 best performing clones as shown by their reactivity on ELISA were selected for expression and purification. The 11 selected nanobodies had good expression in bacterial cells. Good quality of the soluble nanobodies was obtained from the periplasm of the prokaryotic cells after osmotic shock and IMAC purification. Sequence analysis of the selected VHH sequences and phylogenetic tree construction showed that they grouped to four distinct groups. Therefore, a highly diverse immune library was created against the MCMV recombinant coat protein, corroborating the results from other similar studies that report generation of highly diverse immune library against various antigens [19].

Ligand binding assays showed that the nanobodies could bind to and detect MCMV in infected leaf samples. In the present study, we identified nanobodies capable of binding MCMV, a major causal agent for MLN. These nanobodies could offer an effective way for continuous monitoring and detection of MCMV, enabling timely implementation of control measures in farmers’ fields and seed companies. Additionally, they could help regulate the entry and export of contaminated seed materials.

## Conclusions

In the present study, CP‐MCMV was successfully cloned in a vector and efficiently expressed in a bacterial expression system. The molecular weight of the resulting CP‐MCMV protein was 25 kDa. Previous studies have shown MCMV coat protein to be 236 amino acids long [32]. The coat proteins were of good quality and were used for llama immunization. Eleven nanobodies were selected from the immune library through phage display and panning, and these nanobodies could bind to MCMV in infected leaf samples. The results indicate the potential for these nanobodies to be used in ELISA analysis and rapid test flow diagnostics tests.

## Conflicts of interest

The authors declare that they have no conflicts of interest.

### Peer review

The peer review history for this article is available at https://www.webofscience.com/api/gateway/wos/peer‐review/10.1002/2211‐5463.13882.

## Author contributions

FN conceived, designed the study and carried out the experiments on MCMV nanobody expression and purification. OZ designed the MCMV coat protein expression, llama immunization and library generation experiments and also carried them out, GM supervised the study and revised the draft manuscript. GH supervised the study. KDJ designed and wrote the protocol for the maize plant inoculation experiments, which were carried out at ILVO. JG supervised the study and helped manage the experiments. All authors provided critical feedback and helped shape the research, analysis and manuscript.

## Supporting information


**Fig. S1.** MCMV coat protein sequence as retrieved from GeneBank accession number MK491606.

**Fig. S2.** MCMV infected maize plants at 2 weeks.

**Fig. S3.** MONO S 5/5 cation exchange chromatography of MCMV coat protein.

**Table S1.** Absorbance reading of periplasmic ELISA of 95 randomly picked clones from the 2nd and 3rd round of the panning experiments.

## Data Availability

The data supporting the findings of this study are available within the published article, as well as within the articles referenced and the Supporting information.
