# Supplementary figures and images for: Development of nanobodies against the coat protein of maize chlorotic mottle virus
Source: FEBS Open Bio. 2024 Aug 21;14(10):1746–57. doi: 10.1002/2211-5463.13882 (PMC11452299; doi:10.1002/2211-5463.13882)

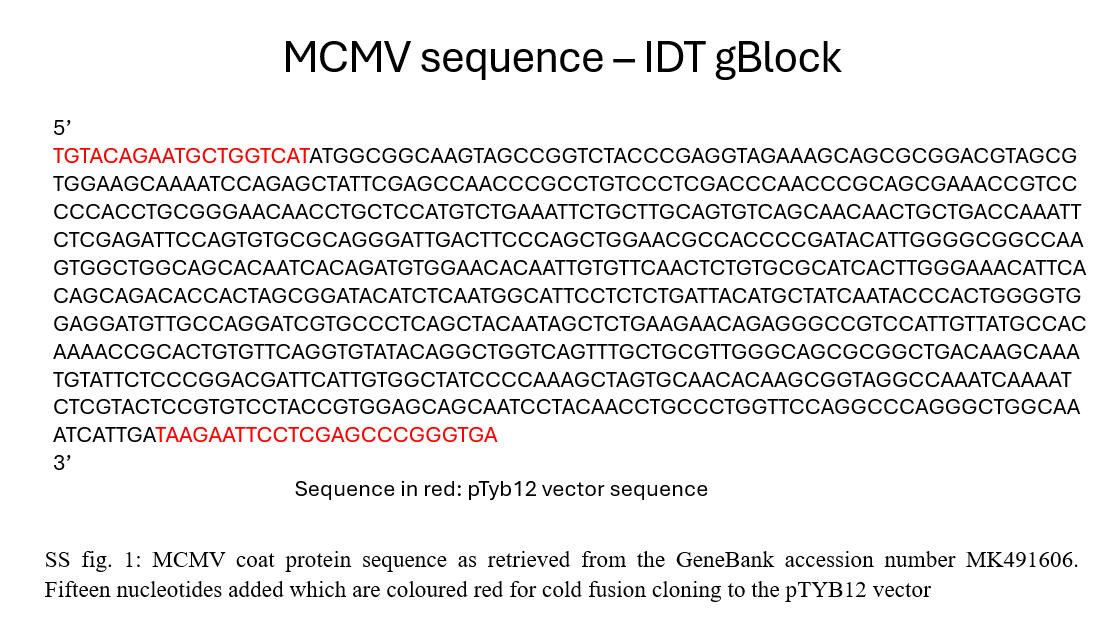

Supplement: Supplementary file 1 — Fig. S1. MCMV coat protein sequence as retrieved from GeneBank accession number MK491606. [file FEB4-14-1746-s004.png]

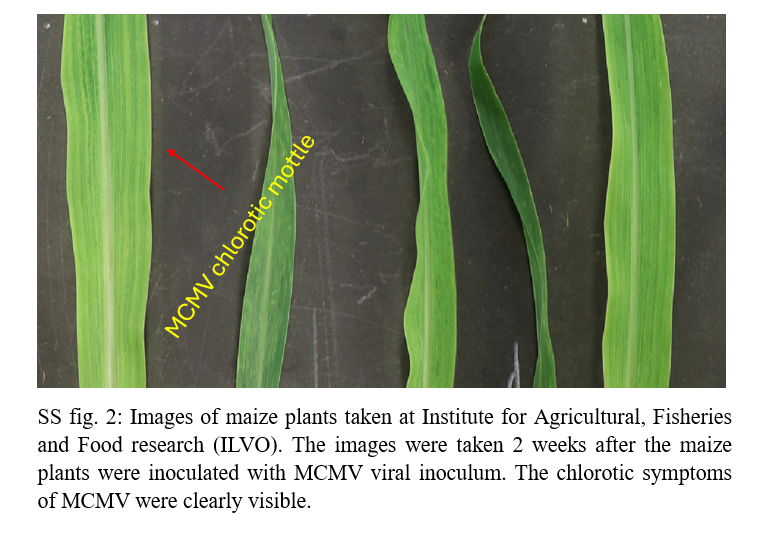

Supplement: Supplementary file 2 — Fig. S2. MCMV infected maize plants at 2 weeks. [file FEB4-14-1746-s001.png]

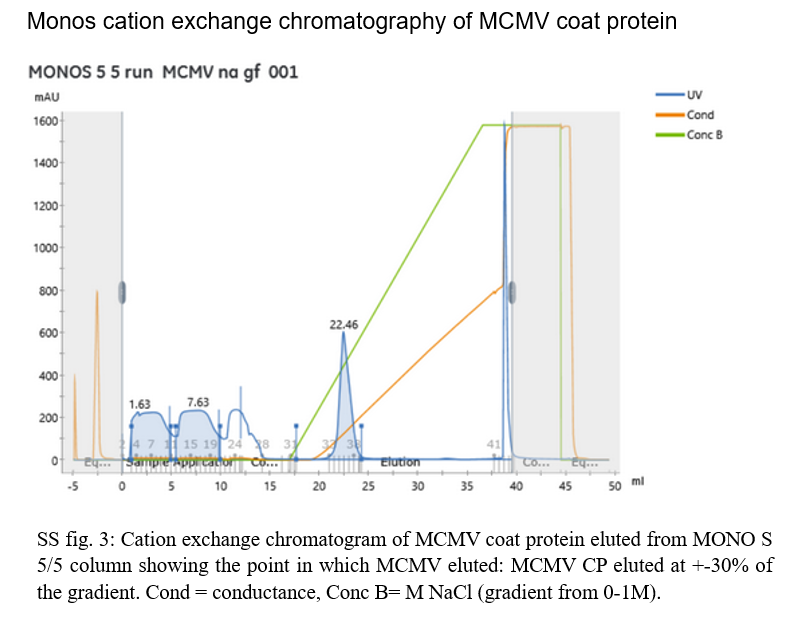

Supplement: Supplementary file 3 — Fig. S3. MONO S 5/5 cation exchange chromatography of MCMV coat protein. [file FEB4-14-1746-s003.png]
