# Supplementary material for: Development of nanobodies against the coat protein of maize chlorotic mottle virus
Source: FEBS Open Bio. 2024 Aug 21;14(10):1746–57. doi: 10.1002/2211-5463.13882 (PMC11452299; doi:10.1002/2211-5463.13882)
Supplement: Supplementary file 4 — Table S1. Absorbance reading of periplasmic ELISA of 95 randomly picked clones from the 2nd and 3rd round of the panning experiments. [file FEB4-14-1746-s002.docx]

Absorbance reading of the results of periplasmic ELISA of 95 randomly picked clones from the 2^nd^ and 3^rd^ round of panning

| **Rank** | | **Clone #** | | **Abs** | | | **Ctl-abs** | | | **ratio** | |
| --- | --- | --- | --- | --- | --- | --- | --- | --- | --- | --- | --- |
| **1** | | 1 | | 0.055 | | | 0.051 | | | 1.1 | |
| **2** | | 2 | | 0.086 | | | 0.057 | | | 1.5 | |
| **3** | | 3 | | 1.183 | | | 0.996 | | | 1.2 | |
| **4** | | 4 | | 0.049 | | | 0.049 | | | 1.0 | |
| **5** | | 5 | | 1.026 | | | 0.051 | | | 20.1 | |
| **6** | | 6 | | 0.285 | | | 0.051 | | | 5.6 | |
| **7** | | 7 | | 0.046 | | | 0.051 | | | 0.9 | |
| **8** | | 8 | | 0.052 | | | 0.052 | | | 1.0 | |
| **9** | | 9 | | 0.17 | | | 0.048 | | | 3.5 | |
| **10** | | 10 | | 2.28 | | | 0.051 | | | 44.7 | |
| **11** | | 11 | | 1.663 | | | 0.056 | | | 29.7 | |
| **12** | | 12 | | 0.603 | | | 0.045 | | | 13.4 | |
| **13** | | 13 | | 0.044 | | | 0.06 | | | 0.7 | |
| **14** | | 14 | | 1.673 | | | 0.049 | | | 34.1 | |
| **15** | | 15 | | 1.223 | | | 0.057 | | | 21.5 | |
| **16** | | 16 | | 0.368 | | | 0.055 | | | 6.7 | |
| **17** | | 17 | | 0.599 | | | 0.071 | | | 8.4 | |
| **18** | | 18 | | 0.035 | | | 0.043 | | | 0.8 | |
| **19** | | 19 | | 1.137 | | | 0.045 | | | 25.3 | |
| **20** | | 20 | | 1.015 | | | 0.073 | | | 13.9 | |
| **21** | | 21 | | 2.065 | | | 0.064 | | | 32.3 | |
| **22** | | 22 | | 2.427 | | | 0.056 | | | 43.3 | |
| **23** | | 23 | | 0.055 | | | 0.048 | | | 1.1 | |
| **24** | | 24 | | 1.185 | | | 0.05 | | | 23.7 | |
| **25** | | 25 | | 0.052 | | | 0.049 | | | 1.1 | |
| **26** | | 26 | | 1.389 | | | 0.049 | | | 28.3 | |
| **27** | | 27 | | 0.059 | | | 0.046 | | | 1.3 | |
| **28** | | 28 | | 0.625 | | | 0.045 | | | 13.9 | |
| **29** | | 29 | | 0.343 | | | 1.058 | | | 0.3 | |
| **30** | | 30 | | 2.088 | | | 0.048 | | | 43.5 | |
| **31** | | 31 | | 1.016 | | | 0.059 | | | 17.2 | |
| **32** | | 32 | | 2.527 | | | 0.058 | | | 43.6 | |
| **33** | | 33 | | 0.14 | | | 0.049 | | | 2.9 | |
| **34** | | 34 | | 0.055 | | | 0.065 | | | 0.8 | |
| **35** | | 35 | | 1.819 | | | 0.049 | | | 37.1 | |
| **36** | | 36 | | 0.311 | | | 0.05 | | | 6.2 | |
| **37** | | 37 | | 0.051 | | | 0.062 | | | 0.8 | |
| **38** | | 38 | | 1.935 | | | 0.087 | | | 22.2 | |
| **39** | | 39 | | 2.048 | | | 0.087 | | | 23.5 | |
| **40** | | 40 | | 2.036 | | | 0.058 | | | 35.1 | |
| **41** | | 41 | | 1.572 | | | 0.058 | | | 27.1 | |
| **42** | | 42 | | 1.896 | | | 0.058 | | | 32.7 | |
| **43** | | 43 | | 0.951 | | | 0.771 | | | 1.2 | |
| **44** | | 44 | | 2.048 | | | 0.052 | | | 39.4 | |
| **45** | | 45 | | 1.863 | | | 0.054 | | | 34.5 | |
| **46** | | 46 | | 1.625 | | | 0.049 | | | 33.2 | |
| **47** | | 47 | | 0.462 | | | 0.064 | | | 7.2 | |
| **48** | | 48 | | 0.048 | | | 0.057 | | | 0.8 | |
| **1** | | 49 | | 2.531 | | | 0.055 | | | 46.0 | |
| **2** | | 50 | | 2.212 | | | 0.075 | | | 29.5 | |
| **3** | | 51 | | 1.762 | | | 0.057 | | | 30.9 | |
| **4** | | 52 | | 2.051 | | | 0.069 | | | 29.7 | |
| **5** | | 53 | | 1.995 | | | 0.078 | | | 25.6 | |
| **6** | | 54 | | 2.083 | | | 0.055 | | | 37.9 | |
| **7** | | 55 | | 1.981 | | | 0.059 | | | 33.6 | |
| **8** | | 56 | | 1.869 | | | 0.056 | | | 33.4 | |
| **9** | | 57 | | 1.615 | | | 0.059 | | | 27.4 | |
| **10** | | 58 | | 0.736 | | | 0.057 | | | 12.9 | |
| **11** | | 59 | | 1.878 | | | 0.051 | | | 36.8 | |
| **12** | | 60 | | 0.203 | | | 0.046 | | | 4.4 | |
| **13** | | 61 | | 1.819 | | | 0.051 | | | 35.7 | |
| **14** | | 62 | | 1.046 | | | 0.052 | | | 20.1 | |
| **15** | | 63 | | 0.251 | | | 0.05 | | | 5.0 | |
| **16** | | 64 | | 1.67 | | | 0.05 | | | 33.4 | |
| **17** | | 65 | | 1.836 | | | 0.05 | | | 36.7 | |
| **18** | | 66 | | 0.954 | | | 0.049 | | | 19.5 | |
| **19** | | 67 | | 1.859 | | | 0.055 | | | 33.8 | |
| **20** | | 68 | | 1.776 | | | 0.053 | | | 33.5 | |
| **21** | | 69 | | 0.161 | | | 0.049 | | | 3.3 | |
| **22** | | 70 | | 1.936 | | | 0.067 | | | 28.9 | |
| **23** | | 71 | | 1.547 | | | 0.054 | | | 28.6 | |
| **24** | | 72 | | 2.28 | | | 0.067 | | | 34.0 | |
| **25** | | 73 | | 2.076 | | | 0.06 | | | 34.6 | |
| **26** | | 74 | | 1.546 | | | 0.047 | | | 32.9 | |
| **27** | | 75 | | 1.637 | | | 0.049 | | | 33.4 | |
| **28** | | 76 | | 1.407 | | | 0.047 | | | 29.9 | |
| **29** | | 77 | | 1.671 | | | 0.049 | | | 34.1 | |
| **30** | | 78 | | 1.528 | | | 0.056 | | | 27.3 | |
| **31** | | 79 | | 1.684 | | | 0.074 | | | 22.8 | |
| **32** | | 80 | | 0.966 | | | 0.054 | | | 17.9 | |
| **33** | | 81 | | 0.171 | | | 0.048 | | | 3.6 | |
| **34** | | 82 | | 1.733 | | | 0.05 | | | 34.7 | |
| **35** | | 83 | | 0.164 | | | 0.059 | | | 2.8 | |
| **36** | | 84 | | 0.426 | | | 0.208 | | | 2.0 | |
| **37** | | 85 | | 2.053 | | | 0.049 | | | 41.9 | |
| **38** | | 86 | | 2.128 | | | 0.04 | | | 53.2 | |
| **39** | | 87 | | 0.285 | | | 0.039 | | | 7.3 | |
| **40** | | 88 | | 1.832 | | | 0.053 | | | 34.6 | |
| **41** | | 89 | | 1.801 | | | 0.05 | | | 36.0 | |
| **42** | | 90 | | 0.478 | | | 0.047 | | | 10.2 | |
| **43** | | 91 | | 1.594 | | | 0.064 | | | 24.9 | |
| **44** | | 92 | | 1.336 | | | 0.065 | | | 20.6 | |
| **45** | | 93 | | 1.37 | | | 0.048 | | | 28.5 | |
| **46** | | 94 | | 0.363 | | | 0.051 | | | 7.1 | |
| **47** | | 95 | | 1.237 | | | 0.049 | | | 25.2 | |
| **bl** | | bl | | 0.044 | | | 0.05 | | | 0.9 | |
|  | |  | |  | |  |  | |  |  |  |
|  | | Abs < 2 | | | |  |  | |  |  |  |
|  | | Abs < 1 | | | |  |  | |  |  |  |
|  | |  | |  | |  |  | |  |  |  |
|  | | Control > 0,3 | | | |  |  | |  |  |  |
|  | | Control > 1 | | | |  |  | |  |  |  |
|  | |  | |  | |  |  | |  |  |  |
|  | | ratio >10 | | | |  |  | |  |  |  |
|  | | ratio > 3 | | | |  |  | |  |  |  |
|  | | ratio > 2 | | | |  |  | |  |  |  |
|  | | ratio < 2 | | | |  |  | |  |  |  |
|  | |  | |  | |  |  | |  |  |  |
|  | | = selected for sequencing | | | | | | |  |  |  |
|  | | automatic selecting happens when: | | | | | | | |  |  |
|  | | 1) ratio higher than selected factor | | | | | | | |  |  |
|  | | 2) Absolute value at least the low-cutoff value | | | | | | | | | |
|  | | 3) Control value lower than the Ctrl-cutoff value | | | | | | | | | |

SS Table 1: Table showing the absorbance reading of the results of periplasmic ELISA of the 95 randomly picked clones from the 2^nd^ and 3^rd^ round of the panning experiments to select for nanobodies against MCMV CP. The absorbance of the antigen coated well was twice that of the control well in 76 of the selected clones.
